# Prominent elevation of extracellular matrix molecules in intracerebral hemorrhage

**DOI:** 10.3389/fnmol.2023.1251432

**Published:** 2023-11-06

**Authors:** Hongmin Li, Samira Ghorbani, Ruiyi Zhang, Vincent Ebacher, Erin L. Stephenson, Michael B. Keough, V. Wee Yong, Mengzhou Xue

**Affiliations:** ^1^Department of Cerebrovascular Diseases, The Second Affiliated Hospital of Zhengzhou University, Zhengzhou, Henan, China; ^2^Academy of Medical Science, Zhengzhou University, Zhengzhou, Henan, China; ^3^Hotchkiss Brain Institute, University of Calgary, Calgary, AB, Canada; ^4^Department of Clinical Neurosciences, University of Calgary, Calgary, AB, Canada; ^5^Department of Cell Biology and Anatomy, University of Calgary, Calgary, AB, Canada; ^6^Department of Pathology and Laboratory Medicine, University of Calgary, Calgary, AB, Canada; ^7^Division of Neurosurgery, University of Alberta, Edmonton, AB, Canada

**Keywords:** extracellular matrix, neuroinflammation, chondroitin sulfate proteoglycans, remyelination, intracerebral hemorrhage

## Abstract

**Background:**

Intracerebral hemorrhage (ICH) is the predominant type of hemorrhagic stroke with high mortality and disability. In other neurological conditions, the deposition of extracellular matrix (ECM) molecules is a prominent obstacle for regenerative processes and an enhancer of neuroinflammation. Whether ECM molecules alter in composition after ICH, and which ECM members may inhibit repair, remain largely unknown in hemorrhagic stroke.

**Methods:**

The collagenase-induced ICH mouse model and an autopsied human ICH specimen were investigated for expression of ECM members by immunofluorescence microscopy. Confocal image z-stacks were analyzed with Imaris 3D to assess the association of immune cells and ECM molecules. Sections from a mouse model of multiple sclerosis were used as disease and staining controls. Tissue culture was employed to examine the roles of ECM members on oligodendrocyte precursor cells (OPCs).

**Results:**

Among the lectican chondroitin sulfate proteoglycan (CSPG) members, neurocan but not aggrecan, versican-V1 and versican-V2 was prominently expressed in perihematomal tissue and lesion core compared to the contralateral area in murine ICH. Fibrinogen, fibronectin and heparan sulfate proteoglycan (HSPG) were also elevated after murine ICH while thrombospondin and tenascin-C was not. Confocal microscopy with Imaris 3D rendering co-localized neurocan, fibrinogen, fibronectin and HSPG molecules to Iba1^+^ microglia/macrophages or GFAP^+^ astrocytes. Marked differentiation from the multiple sclerosis model was observed, the latter with high versican-V1 and negligible neurocan. In culture, purified neurocan inhibited adhesion and process outgrowth of OPCs, which are early steps in myelination *in vivo*. The prominent expression of neurocan in murine ICH was corroborated in human ICH sections.

**Conclusion:**

ICH caused distinct alterations in ECM molecules. Among CSPG members, neurocan was selectively upregulated in both murine and human ICH. In tissue culture, neurocan impeded the properties of oligodendrocyte lineage cells. Alterations to the ECM in ICH may adversely affect reparative outcomes after stroke.

## 1. Introduction

ICH is a devastating disorder characterized by formation of hematoma within the brain parenchyma, with possible blood extension into the ventricles (Qureshi et al., 2001; Ziai and Carhuapoma, 2018). ICH accounts for 15–20% of all strokes, with a disability rate of 70% and a mortality rate of over 50% annually (van Asch et al., 2010). The poor recovery of ICH generally results from the ongoing toxic neuroinflammation following the mechanical disruption of extravasated blood (Bai et al., 2020; Xue and Yong, 2020). Recovery may also be affected by brain ECM components that impact inflammation and repair following other types of central nervous system (CNS) injury (Stephenson et al., 2019; Ghorbani et al., 2022).

The ECM is an intricate network of proteins and sugars dispersed throughout the extracellular space of the CNS (Stephenson and Yong, 2018). The ECM can be segregated into the basement membrane around the cerebral microvessels, interstitial matrix between cellular structures in the parenchyma, and perineuronal nets around certain neuronal soma (Miller, 2017). The major components of the ECM with known roles in injury and repair include chondroitin sulfate proteoglycans (CSPGs), fibrinogen, fibronectin, heparan sulfate proteoglycans (HSPGs), and thrombospondins (TSPs).

CSPGs are widely expressed in the CNS. They consist of a central core protein and varying number of glycosaminoglycan (GAG) side chains (Stephenson and Yong, 2018). The lectican CSPG subfamily include aggrecan, brevican, neurocan and versican (Zimmermann and Ruoslahti, 1989). Versicans have five splice isoforms: V0 (containing both GAG-beta and alpha domains), V1 (only GAG-beta domain), V2 (only GAG-alpha domain), V3 (neither GAG-beta nor alpha domains), and V4 (part of GAG-beta domain) (Kischel et al., 2010). Fibrinogen, a blood-derived soluble protein synthesized by hepatocytes (Liebner et al., 2011) enters the CNS parenchyma and is converted into insoluble fibrin in CNS injury with blood–brain barrier disruption (Petersen et al., 2018). Fibronectin exists as a protein dimer through two disulfide bridges and acts as a scaffold for adhering cells, binding heparan and collagen (George et al., 1993). Heparan sulfate proteoglycans (HSPGs) are found in basement membrane, and are comprised of a core protein with attached heparan sulfate GAGs (Kjellén and Lindahl, 1991). Thrombospondins (TSPs) are a family of calcium-binding ECM glycoproteins that regulate cell–cell and cell-matrix interactions (Ponticelli and Anders, 2017). Tenascin-C is another glycoprotein that is commonly elevated in CNS lesions (Roll and Faissner, 2019).

The ECM is essential for homeostasis and serves metabolic and structural functions in the CNS. However, ECM components are deposited and remodeled aberrantly in neurological diseases and this affects both injury and repair (Stephenson and Yong, 2018). For example, various ECM members including versican-V1, fibrinogen, fibronectin, HSPGs and TSP are elevated at the peak of clinical disability in an animal model of multiple sclerosis (Stephenson et al., 2018; Ghorbani and Yong, 2021; Ghorbani et al., 2022). Sources of ECM deposition after injury includes astrocytes, oligodendrocytes, neurons, pericytes and endothelial cells (Stephenson et al., 2018). In chronic multiple sclerosis lesions, the expression of lectican CSPGs including aggrecan and versican rises (Sobel and Ahmed, 2001; Stephenson et al., 2019). Following lysolecithin-induced demyelination, another model of multiple sclerosis, the level of versican-V1 is elevated in lesions (Keough et al., 2016).

In ICH, demyelination occurs in the perihematomal area and lesion core, beginning by 1 day after injury, peaking at 3 days, and it is not ongoing at 28 days (Moxon-Emre and Schlichter, 2011; Fu et al., 2021). The possible mechanisms involved in demyelination in ICH include mechanical injury induced by the edema and hematoma that compresses white matter fibers (Tao et al., 2017; Zhu et al., 2018). Furthermore, oxidative stress, ischemic damage caused by prolonged edema, blood–brain barrier disruption, neuroinflammation and glutamate excitotoxicity exacerbate the demyelination (Matute et al., 2007; Hu et al., 2016; Martinez Sosa and Smith, 2017).

There is currently a gap of knowledge on the expression and functions of ECM components in ICH (Li et al., 2023). After intraventricular hemorrhage in premature rabbit pups, levels of CSPGs are elevated in the forebrain (Vinukonda et al., 2013). As pro-inflammatory macrophages can produce versican-V1 (Toeda et al., 2005; Stephenson et al., 2018) and as chronic CSPG deposition inhibits repair processes after spinal cord injury (Properzi et al., 2003; Rolls et al., 2008), it is worth considering whether particular CSPG members are elevated after ICH to impede attempts at regeneration. Moreover, as versican-V1 promotes cytotoxic neuroinflammation in a model of multiple sclerosis (Ghorbani et al., 2022), and CSPGs enhance the production of ICH-associated matrix metalloproteinases (MMPs) and pro-inflammatory cytokines (Sun et al., 2023), it is imperative to determine whether the ECM is dysregulated in ICH.

In this study, we examined whether and which ECM components are altered after ICH, with focus in the perihematomal area and lesion core. We determined which cell type expresses the elevated ECM molecules, and whether a particular ECM molecule is potentially inhibitory to OPCs in ICH. These results were contrasted with specimens from experimental autoimmune encephalomyelitis (EAE), an animal model of multiple sclerosis used as positive controls for antibody staining. Our overall objective is to derive new insights in whether the ECM is a novel target to improve prognosis after ICH.

## 2. Methods

All animal experiments were performed with ethics approval (protocol number AC21-0073) from the Animal Care Committee at the University of Calgary under regulations of the Canadian Council of Animal Care.

### 2.1. Mice

Male C57BL/6 wildtype mice (8–12 weeks old) for ICH were purchased from Charles River. C57BL/6 wildtype mice (8–12 weeks old) for EAE were acquired from Jackson Laboratories. Litters from pregnant CD1 mice (P1-P2) were purchased from Charles River and used for OPC cultures. CX3CR1^CreER^:Rosa26^TdT^ (Ai9) mice at 8–10 weeks were injected intraperitonially with 2 mg tamoxifen (100 μL of 20 mg/mL tamoxifen in corn oil) (Sigma) daily for 3 consecutive days to induce recombination. Four weeks later, allowing time for fast-turnover Td Tomato+ (TdT+) monocytes to be replaced by wildtype cells so that Iba1 + TdT+ cells in the injured CNS would be microglia rather than monocyte-derived macrophages (Plemel et al., 2020), ICH was inflicted as described below. Genotyping was conducted using protocol provided online through Jackson Laboratories. Mice were housed at 22°C, with 12 h light and 12 h dark cycle, environmental enrichment and free access to food and water.

### 2.2. ICH induction

The protocol for induction has been described elsewhere (Zhang et al., 2023). In brief, 0.05 U of collagenase type VII dissolved in 0.5 μL of saline was injected at a rate of 0.1 μL/min over 5 min into the right striatum. Then, the needle was maintained at the same spot for additional 5 min to prevent reflux. The mice were sutured and then monitored in a thermally controlled environment until recovery.

### 2.3. EAE induction and tissue harvest

Mice were injected subcutaneously with 200 μg MOG35-55 peptide emulsified in complete Freund’s adjuvant containing 10 mg/mL of heat inactivated *Mycobacterium tuberculosis* H37RA as described elsewhere (Ghorbani et al., 2022). On the day of immunization and 48 h later, animals received intraperitoneal injections of 300 ng of pertussis toxin (List Biological Laboratories). Spinal cord sections from mice killed at peak clinical severity (around day 18 after MOG) were used for immunofluorescence analyses.

### 2.4. ICH brain tissue harvest

Mice were sacrificed at 7-days post-collagenase injection with a lethal dose of ketamine and xylazine. Animals were perfused with a total of 10 mL of phosphate-buffered saline (PBS) and 10 mL of 4% paraformaldehyde (PFA) in PBS via cardiac puncture. The whole brain was collected into 4% PFA in PBS for fixation overnight, and then was transferred into 30% sucrose solution for 72 h. The cerebellum was excised, and the remaining brain tissue was frozen in FSC 22 frozen section media (Leica). Brain blocks were cut coronally by a cryostat into 20 μm sections, collected onto microscope slides and stored at −20°C before staining.

### 2.5. Human ICH specimen

Paraffin-embedded blocks from an autopsied ICH subject was from Foothills Medical Center, University of Calgary. The subject was a 70-year-old male who had a right middle cerebral artery (MCA) infarct 5 days before death, and with a hemorrhagic transformation 2 days after. Thus, death occurred 3 days after ICH. Sample was collected with full informed consent for autopsy and retention of tissues for research. Gross examination revealed a substantial hemorrhage in the right cerebral parenchyma, along with edema and gyral flattening. The perihematomal regions (because the main hemorrhage region was purely hemorrhage and necrotic tissue) and the contralateral side containing both frontal white matter and cortical regions were taken for research. The use of these human tissues in Calgary for research was approved by the Conjoint Health Research Ethics Board at the University of Calgary (Ethics ID REB15-0444).

### 2.6. Antibodies

The primary and secondary antibodies used are displayed in Table 1.

**Table 1:** Primary and secondary antibodies.

Target	Antibody	Commercial source	Catalog number	Dilution from supplier stock
Astrocytes	Chicken anti-mouse glial fibrillary acidic protein (GFAP)	BioLegend	829,401	1:1,000
Microglia/macrophages	Goat anti-human/mouse Iba1	ThermoFisher	PA5-18039	1:250
Neuron	Rabbit anti-human/mouse NeuN	Abcam	AB 177487	1:500
Leukocytes	Rat anti-human/mouse CD45	ThermoFisher	MA5-17687	1:50
Versican-V1	Rabbit anti-mouse versican V0/V1	Millipore	AB 1033	1:100
Versican-V2	Rabbit anti-mouse versican V0/V2	Millipore	AB 1032	1:100
Neurocan	Rabbit anti-mouse neurocan	Millipore	ABT 1347	1:100
Fibrinogen	Rabbit anti-human/mouse fibrinogen	Abcam	AB 34269	1:100
Fibronectin	Rabbit anti-human/mouse fibronectin	Abcam	AB 23750	1:100
Thrombospondin-1	Rabbit anti-human/mouse thrombospondin-1	Abcam	AB 85762	1:100
Tenascin-C	Rat anti-human/mouse tenascin C	R&D	MAB2138	1:50
Aggrecan	Rabbit anti-mouse aggrecan	Millipore	AB 1031	1:100
HSPG	Mouse anti-mouse heparan sulfate proteoglycan	Amsbio	370,255	1:100
CSPG	Mouse anti-mouse CSPG	Millipore	MAB2030	1:100
Oligodendrocyte	Mouse anti-human/mouse oligodendrocyte marker O4	R&D	MAB1326	1:50
Oligodendrocyte	Rabbit anti-human/mouse olig-2	Millipore	AB 9610	1:200
PDGFRα	Goat anti-mouse PDGFR alpha	R&D	AF 1062	1:200
Isotype	Rabbit isotype control	Abcam	AB 37415	1:100
Nuclear	DAPI	ThermoFisher	62,248	1:800
Rabbit IgG	Alexa Fluor 647 donkey anti-rabbit IgG	Jackson ImmunoResearch	711–605-152	1:400
Goat IgG	Alexa Fluor 488 donkey anti-goat IgG	Jackson ImmunoResearch	705–545-147	1:400
Mouse IgM	Alexa Fluor 488 donkey anti-mouse IgM	Jackson ImmunoResearch	715–545-140	1:400
Chicken IgY	Cyanine Cy3 donkey anti-chicken IgY	Jackson ImmunoResearch	703–165-155	1:400
Goat IgG	Cyanine Cy3 donkey anti-goat IgG	Jackson ImmunoResearch	705–165-147	1:400

### 2.7. Immunofluorescence staining

Microscope slides containing mouse brain tissues were thawed at room temperature for 30 min, then hydrated with PBS for 5 min, and permeabilized with 0.1% Triton X-100 in PBS for 5 min. Tissue sections were blocked by horse serum blocking solution (0.01 M PBS, 1% bovine serum albumin (BSA), 10% horse serum, 0.1% Triton-X100, 0.1% cold fish gelatin, and 0.05% Tween-20) for 1 h. Alternatively, for staining using the HSPG antibody in mice, AffiniPure Fab fragment donkey anti-mouse IgG (H + L) (Jackson ImmunoResearch, 715–007-003, 1:50) was added to the blocking buffer. Tissues were incubated with primary antibodies suspended in antibody dilution buffer (0.01 M PBS, 1% BSA, 0.1% Triton-X100, 0.1% cold fish gelatin) overnight at 4°C. Next, slides were washed with PBS containing 0.2% Tween-20 and incubated with fluorophore conjugated secondary antibodies (1,400) and 1 μg/mL of DAPI for 1 h. The slides were washed and mounted using Fluoromount-G solution (SouthernBiotech).

For CSPG staining, chondroitinase ABC (ChABC, Sigma) digestion was performed prior to the blocking step in order to remove GAG chains to facilitate antibody binding to the core protein. Slides were then incubated with ChABC diluted in PBS (0.2 U/mL) for 30 min at 37°C.

For human paraffin sections, these were cut (7 μm) using a Leica RM2135 Microtome. Following deparaffinization, sections were subjected to antigen retrieval by boiling in 10 mM sodium citrate buffer (pH 6.0) for 20 min, then enzymatically digested with ChABC at 37°C for 30 min. The sections were next incubated with 4% horse serum to block nonspecific binding, then incubated with anti neurocan, anti-GFAP and anti-Iba1 overnight at 4°C. Secondary antibodies (Table 1) were added for 60 min. Fluorescence images were taken on a confocal microscope (Fluoview FV10i; Olympus).

### 2.8. Cell culture

#### 2.8.1. Mouse oligodendrocyte precursor cells

Brains from postnatal day P0-2 mouse pups were isolated and processed for OPCs as described previously (Keough et al., 2016; Ghorbani et al., 2022). OPCs were seeded at a density of 1 × 10^4^ cells per well of 96-well flat bottom black plates in 100 μL of oligodendrocyte differentiating medium (Keough et al., 2016). Note that all wells were pre-coated with poly-l-lysine (10 μg/mL) prior to OPCs being added, as our experience is that uncoated plastic is an unreliable substrate for adhesion of OPCs resulting in huge variability of attached cells across non-coated wells. To test the effects of ECM molecules on OPCs, the poly-L-lysine pre-coated wells were exposed to poly-L-lysine (10 μg/mL, control), neurocan (10 μg/mL, Millipore), a mixed CSPG preparation (10 μg/mL, Millipore, and containing neurocan, aggrecan, versican and phosphacan according to the supplier), fibronectin (10 μg/mL, Sigma) or fibrinogen (10 μg/mL, Millipore) for 3 h at room temperature. A limitation here is that we did not assess whether equivalent amounts of the different ECM molecules had attached onto the poly-L-lysine coating. Following a wash, OPCs were added and grown at 37°C and 8.5% CO2 for 24–72 h. Culture medium is described elsewhere (Sobel and Ahmed, 2001; Keough et al., 2016). The purity of olig2+ oligodendrocyte lineage cells was over 80%.

In an additional experiment, the mixed CSPG preparation was incubated with chondroitinase ABC (Sigma C3667, 0.1–1.0 U/mL) overnight at 37°C, to remove the glycosaminoglycan chains of CSPGs, before coating of wells. Bovine serum albumin (BSA) was also digested with the same enzyme as a control.

For immunocytochemistry, OPCs were fixed for 10 min at RT using 4% paraformaldehyde, rinsed with PBS and then permeabilized with 0.2% Triton X-100 for 10 min at room temperature. The primary antibody mouse anti-mouse sulfatide O4 (oligodendrocyte lineage cell marker) (R&D) was applied overnight at 4°C, followed by secondary antibody and DAPI. Cells were analyzed with a Molecular Devices ImageXpress Micro XLS (Sobel and Ahmed, 2001).

### 2.9. Widefield and confocal fluorescence microscopy

Overviews of brain sections were imaged with an Olympus VS120 slide scanner using a 10×/0.4NA objective. These were used to locate the lesions. Only the brightness and contrast were adjusted to better display representative images. All samples were then imaged on a Leica TCS SP8 laser scanning confocal microscope using a 25×/0.95 NA water objective. Three-dimensional (3D) z-stacks images (2048 × 2048 × 37 voxels) of the four fluorescent probes were acquired using the 405, 488, 552, and 640 nm lasers sequentially at either 228 × 228 × 567 nm or 114 × 114 × 567 nm voxel size. The two different resolutions were used for quantifying % area of ECM molecules in Regions of Interest (ROIs) and % positive cells with ECM molecules, respectively. Imaging parameters were kept constant for each set of experiments.

### 2.10. Confocal image analysis

ImageJ software (NIH) was used to quantify the % ECM molecules in ROIs. For each z-stack image, maximum-intensity projections were created and ROIs were drawn around the perihematomal, lesion center, and contralateral areas according to GFAP or Iba1 labeling. Image segmentation was achieved using a set intensity threshold for each probe. Negative secondary antibody controls or contralateral controls were used to assess baseline signals and determine thresholds. For each probe, intensity threshold as well as size and circularity filters were kept constant across all samples for each experimental set. Total area and percent area (i.e., % ECM molecules in ROI) were measured.

Imaris software (Oxford Instruments) was used to determine the percentage of Iba1^+^ or GFAP^+^ cells overlapping with ECM molecules. Labeled areas were segmented as surfaces via a set intensity threshold determined as above. Iba1^+^ or GFAP^+^ cells were separated using seed points within the Imaris Surface creation workflow to get the total number of cells. For each probe, intensity threshold, surface details were kept constant within each set of experiments. % positive cells with ECM molecules were obtained by dividing the number of positive cells with ECM molecule by the total number of positive cells.

For better visualization of representative images shown, brightness and contrast were adjusted consistently across all samples and the images were converted to RGB.

### 2.11. ImageXpress acquisition and MetaXpress analysis

Labeled cells in 96-well flat bottom black/clear plates (Falcon 353219) were imaged with a 20×/0.45 NA objective on a Molecular Devices ImageXpress Micro XLS. For each well, 12 images (field of views; FOVs) were acquired and analyzed with Molecular devices MetaXpress software. The “Multiwavelength cell scoring” module was used to quantify the cell survival and also cell number for a particular marker. OPC outgrowth was measured using the “Neurite outgrowth” module, which quantifies the processes of cells. Data from the 12 images were averaged to a single data point per well, with seven well replicates per group. The number of surviving cells in each sample was then divided by the mean of the control samples to obtain the fold-change value. For better visualization of representative images shown, brightness and contrast were adjusted consistently across all samples.

### 2.12. Statistics

Microsoft Excel (Version 2022 Build 16.69.1) was used for collating data. All graphs were generated using GraphPad Prism 9.3.1. Shapiro–Wilk normality test was applied to verify normal distribution of data. One-way analysis of variance (ANOVA) with Tukey’s multiple comparison test was used to analyze statistically significant differences among multiple groups. Unpaired two-tailed Student’s *t*-tests was used to compare two groups. *p* < 0.05 was considered statistically significant. All values are shown as mean ± SEM.

## 3. Results

### 3.1. Upregulation of neurocan at perihematomal area and lesion core in murine ICH

The murine ICH lesions were assessed by Hematoxylin & Eosin (H&E) staining at days 3, 7, and 14 after collagenase-induced injury. We found the hematoma size to decrease over time (Supplementary Figure S1A), likely as a result of phagocytosis of erythrocytes and debris by microglia/macrophages and the resolution of edema as reviewed elsewhere (Bai et al., 2020; Xue and Yong, 2020). For detailed examinations, we began with lesions at day 7 after ICH, given a sizable lesion and also the accumulation of ECM molecules in the first week in other models of CNS injury (Rolls et al., 2008; Keough et al., 2016). Also, at day 7 of murine ICH, there was prominent accumulation of CD45+ immune cells, Iba1^+^ microglia/macrophages and GFAP^+^ astrocytes, and surviving NeuN^+^ neurons at the perihematomal area (Supplementary Figures S1B–E).

**Figure 1:**
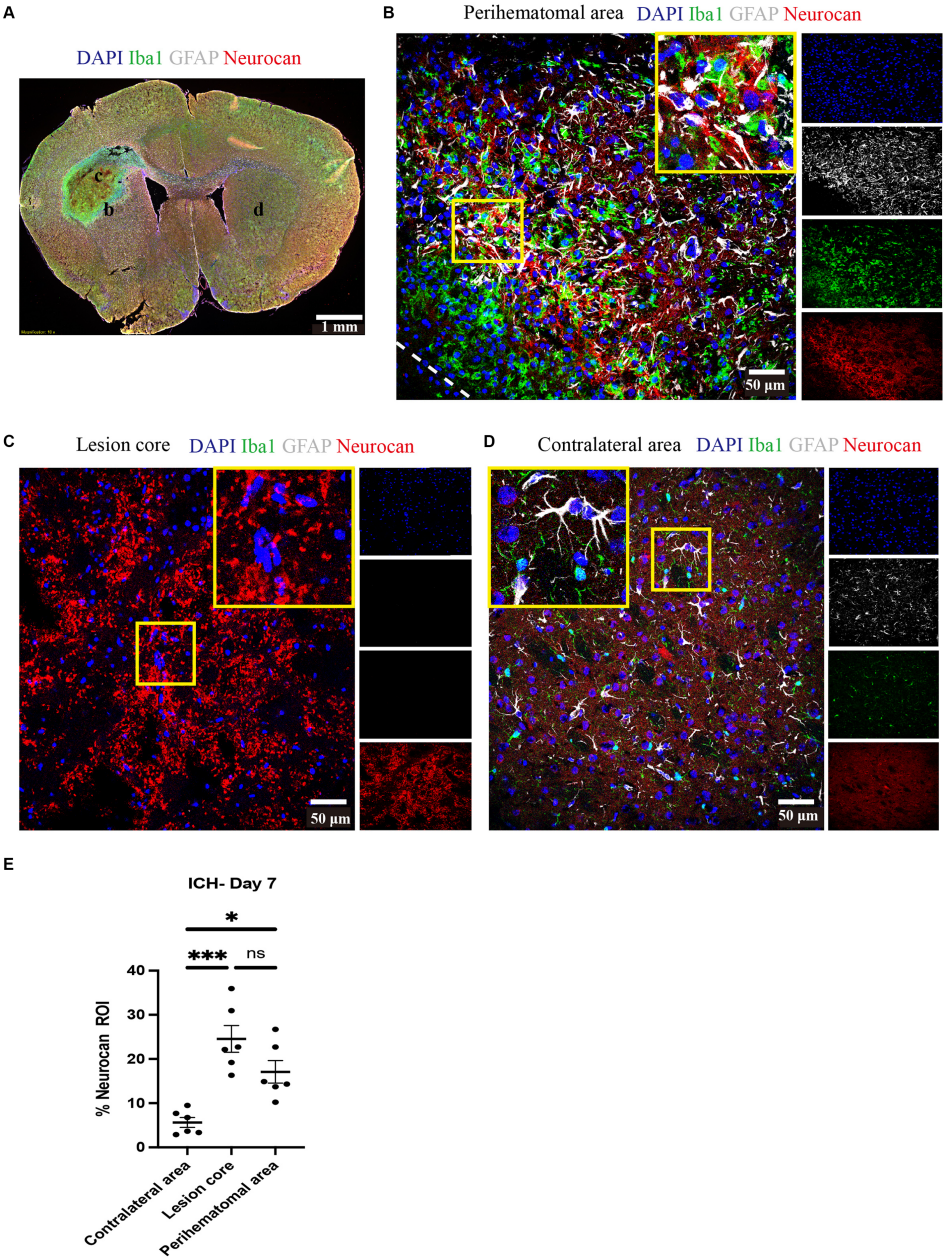
Neurocan is upregulated in perihematomal area and lesion core in murine ICH. **(A)** Representative slide scanner image of coronal brain sections from 7-day ICH mice shows DAPI for cell nuclei (blue), Iba1 for microglia/macrophages (green), GFAP for astrocytes (gray), and neurocan (red) in the perihematomal area (b), lesion core (c) or contralateral area (d). Scale bar = 1 mm. **(B–D)** Representative confocal images from 7-day ICH mice in perihematomal area **(B)**, lesion core **(C)** and contralateral area **(D)** stained for DAPI (blue), Iba1 (green), GFAP (gray), and neurocan (red). The individual colors are represented as individual boxes on the right while the merged image is to the left. The lower left corner inside the dotted lines in **(B)** is the lesion core. The yellow box in upper right/left corner indicates a higher magnification of the area within the smaller box. Scale bar = 50 μm. **(E)** Bar graphs comparing the levels of neurocan in perihematomal area, lesion core and contralateral area at day 7 of ICH, expressed as neurocan ^+^ in region of interest (ROI), where each ROI is a region defined by area occupied by Iba1^+^ cells (*n* = 24 ROIs from 6 mice per group). Data are presented as the mean ± SEM, and analyzed by one-way ANOVA-Tukey’s *post-hoc* test; ns: not significant. Significance indicated as *p < 0.05, ***p < 0.001.

Brain tissue sections at day 7 of ICH were evaluated for the relative elevation of CSPG members. The perihematomal area contained a high density of microglia/macrophages and reactive astrocytes, as reported by Iba1 and GFAP, respectively (Figure 1). In the perihematomal area and lesion core, but not in the contralateral uninjured hemisphere, neurocan immunoreactivity was prominent (Figure 1). Quantitative analysis shows that the elevation of neurocan was comparable between the perihematomal and lesion core at day 7 of ICH (Figure 1E).

No neurocan signal was observed when an isotype antibody control used in place of the primary rabbit neurocan antibody, or omission of neurocan antibody (secondary antibody control) was employed (Supplementary Figure S2).

**Figure 2:**
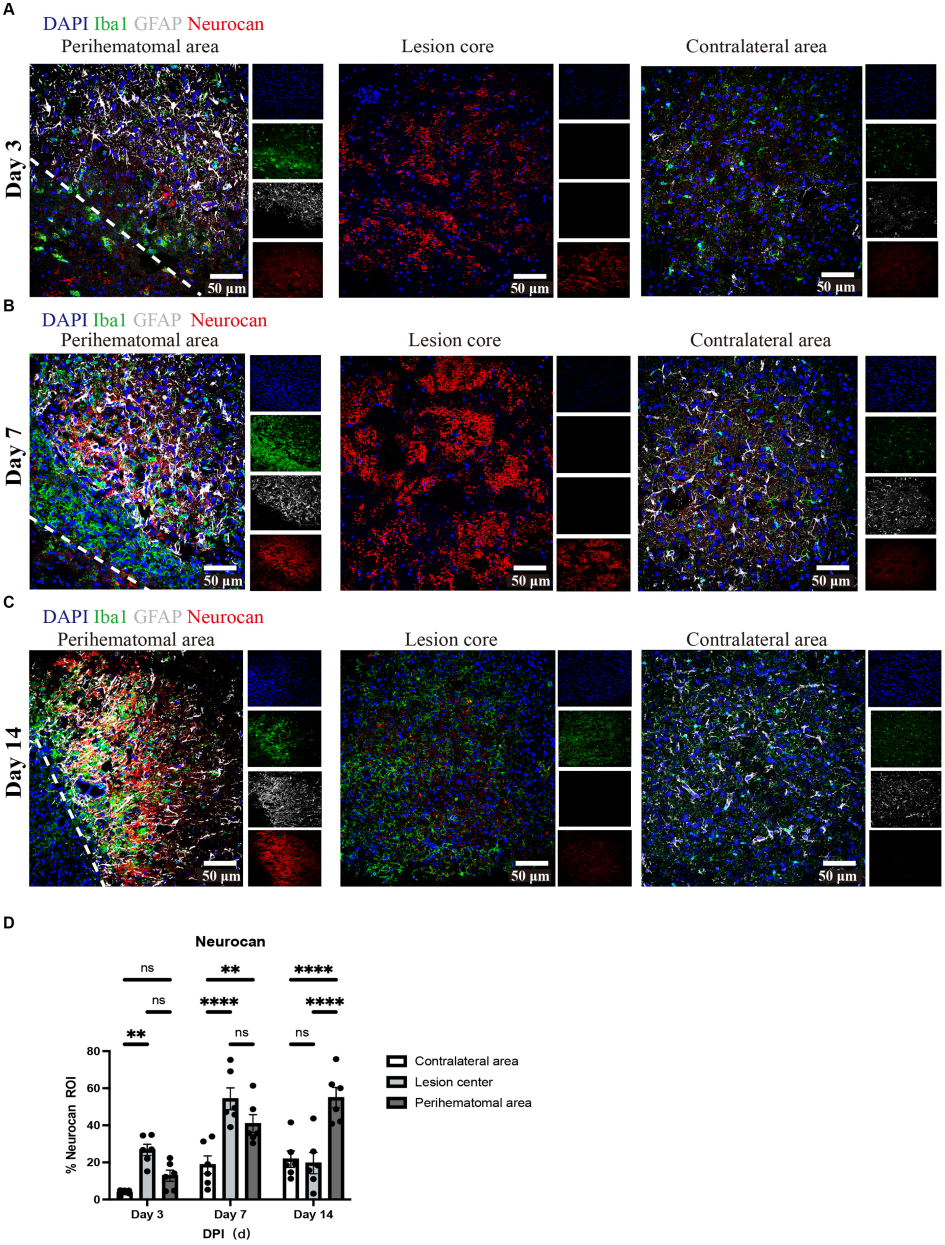
Neurocan expression peaks at day 7 in lesions of murine ICH. **(A–C)** Representative confocal images from 3-day **(A)**, 7-day **(B)** and 14-day **(C)** ICH mice in perihematomal area, lesion core and contralateral area stained for DAPI (blue), Iba1 (green), GFAP (gray), and neurocan (red). The lower left corner inside the dotted lines is the lesion core of ICH. Scale bar = 50 μm. **(D)** Bar graphs comparing the levels of neurocan in perihematomal area, lesion core and contralateral area at three time points, expressed as neurocan ^+^ in region of interest (ROI), where each ROI is a region defined by area occupied by Iba1^+^ cells (*n* = 24 ROIs from 6 mice per group). Data are presented as the mean ± SEM, and analyzed by two-way ANOVA-Tukey’s multiple comparison test; ns, not significant. Significance indicated as **p < 0.01, ****p < 0.0001.

Next, we investigated the levels of neurocan at days 3, 7, and 14 after ICH. Analysis shows detectable expression of neurocan CSPG at day 3 after ICH, a peak at day 7 in the lesion core and perihematomal area and remaining high at day 14 in the latter region of potentially salvageable tissue (Figure 2).

**Figure 3:**
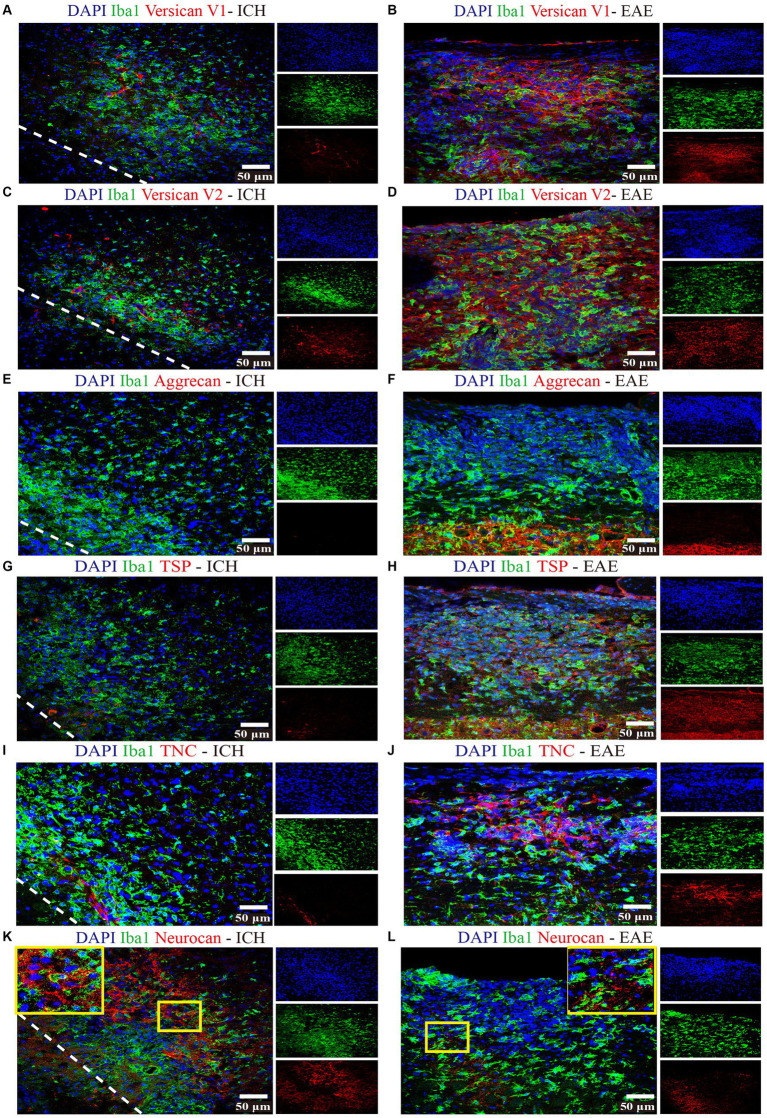
Non-neurocan CSPG members, tenascin-C and thrombospondin, are minimal in ICH while robustly expressed in EAE lesions Representative confocal images of sections from ICH mice at perihematomal area (left) and sections of spinal cord white matter from EAE mice (right). Stains are indicated by the respective colored labels. Images show versican-V1 **(A,B)**, versican-V2 (red) **(C,D)**, aggrecan (red) **(E,F)**, thrombospondin (TSP) (red) **(G,H)**, tenascin-C (TNC, red) **(I,J)**, and neurocan (red) **(K,L)**. The yellow box in upper right/left corner indicates a higher magnification of the area within the smaller box. Versican-V1, V2, TSP, and TNC molecules are accumulated in EAE lesions but not in perihematomal area of ICH. The expression levels of aggrecan are low in both ICH and EAE lesions. However, neurocan aggregates in the perihematomal area of ICH but not in EAE lesions. The lower left corner inside the dotted lines is the lesion core of ICH. Scale bar = 50 μm.

Focusing on day 7 when neurocan expression in lesion is prominent, we addressed the potential elevation of other lectican CSPG members. Unlike neurocan, immunoreactivity was not evident for versican-V1, versican-V2 or aggrecan in ICH, while these CSPGs were found in the positive control EAE tissue (Figure 3). Further contrasting ICH and EAE, the high neurocan immunoreactivity in ICH tissue was negligible in EAE (Figure 3).

### 3.2. Expression of other ECM components in murine ICH

**Figure 4:**
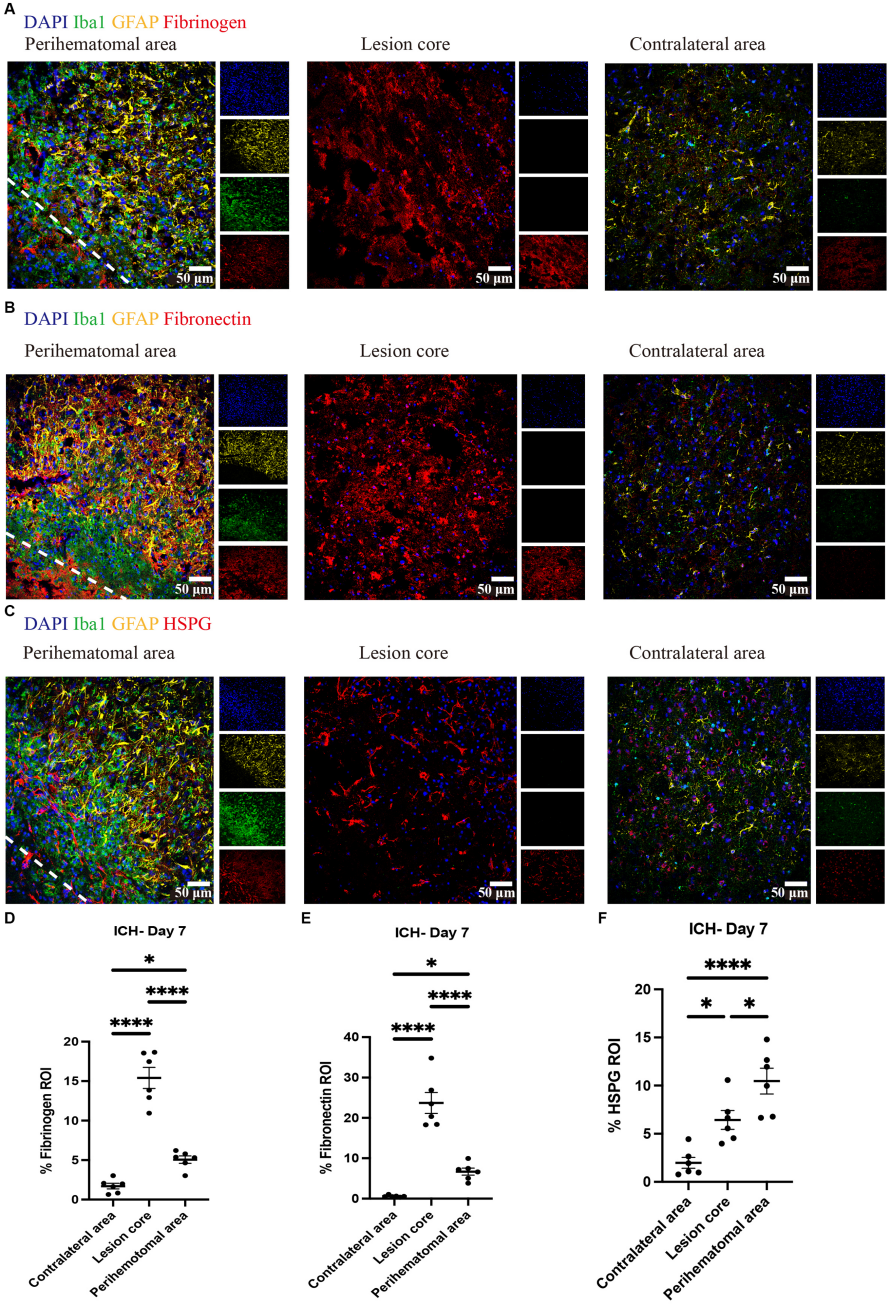
Fibrinogen, fibronectin and HSPG are elevated in perihematomal area and lesion core in ICH mouse model Representative confocal images of brain sections from ICH mice at perihematomal area, lesion core and contralateral area at day 7 stained for DAPI for cell nuclei (blue), Iba1 (green), GFAP (yellow), and fibrinogen (red) **(A)**, fibronectin (red) **(B)** or HSPG (red) **(C)**. Scale bar = 50 μm. **(D–F)** Quantification shows the levels of ECM in perihematomal area and lesion core compared to contralateral area at day 7. *N* = 6 replicates for each group; each dot represents mean of 4 locations of each area analyzed per mouse. Data are presented as the mean ± SEM. One-way ANOVA-Tukey’s *post-hoc* test. Significance indicated as *p < 0.05, ****p < 0.0001.

We evaluated and found thrombospondin (TSP) and tenascin-C (TNC) to be minimally expressed at day 7 of ICH while highly deposited in EAE (Figure 3). Fibrinogen was present in the perihematomal and lesion core areas, albeit in higher amounts in the latter (Figures 4A,D). This was also the case for fibronectin (Figures 4B,E). HSPG was noted in the perihematomal area and lesion core (Figures 4C,F), with a trend toward higher expression in the perihematomal area.

### 3.3. Cellular expression of ECM members that are elevated in murine ICH

**Figure 5:**
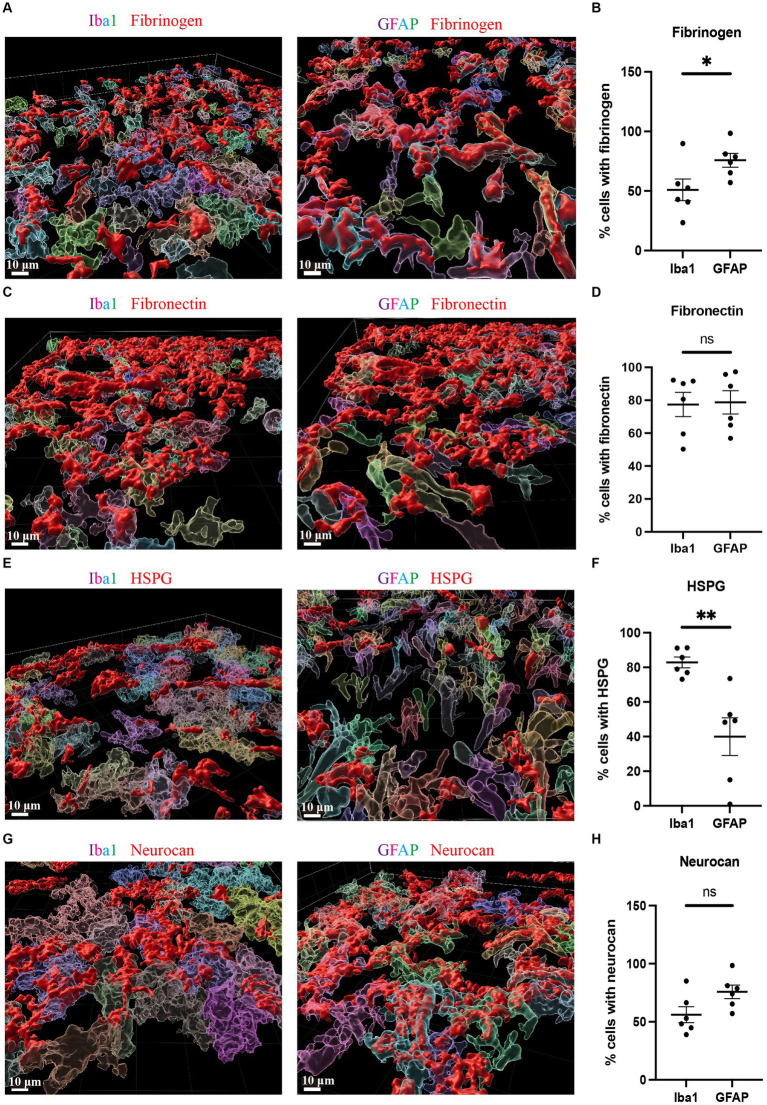
Fibrinogen, fibronectin, HSPG and neurocan within Iba1^+^ or GFAP^+^ cells Representative 3D reconstruction images of perihematomal area at day 7 in ICH using Imaris rendition, showing internal accumulation of fibrinogen (red) **(A)**, fibronectin (red) **(C)**, HSPG (red) **(E)**, or neurocan (red) **(G)** within Iba1^+^ (left) or GFAP^+^ (right) cells. To determine the total count of Iba1^+^ or GFAP^+^ cells, as well as the number of Iba1^+^ or GFAP^+^ cells containing ECM molecules, each cell was individually labeled with a distinct color. Scale bar = 10 μm. We note that the fibrinogen, fibronectin, HSPG and neurocan staining is within Iba1^+^ or GFAP^+^ cells, and also in the extracellular space. **(B,D,F,H)** Quantification showing the percentage of Iba1^+^ or GFAP^+^ cells containing ECM molecules. Data are presented as the mean ± SEM of 6 mice. Each dot represents mean of 4 locations of each area analyzed per mouse. Unpaired two-tailed Student’s *t*-test; ns, not significant. Significance indicated as *p < 0.05, **p < 0.01.

Due to the high accumulation of neurocan, fibrinogen, fibronectin and HSPG in ICH, it was challenging to determine their precise localization within cell types. We thus utilized the 3D surface rendering of the Imaris program to better investigate the association of cells with these ECM molecules. The z-stack surface rendering of Iba1, GFAP and ECM molecules suggested that neurocan, fibrinogen, fibronectin and HSPG were intimately related intracellularly in both Iba1^+^ microglia/macrophages and GFAP^+^ astrocytes, as well as in proximity to these cells in the extracellular space (Figure 5). There appears to be a higher internal accumulation of fibrinogen within GFAP^+^ compared to Iba1^+^ cells (Figure 5B), and higher HSPG signal within Iba1^+^ compared to GFAP^+^ cells (Figure 5F). Neurocan and fibronectin had a similar level of expression within GFAP^+^ and Iba1^+^ cells.

### 3.4. Microglia versus monocyte-derived macrophages in the perihematomal aera

**Figure 6:**
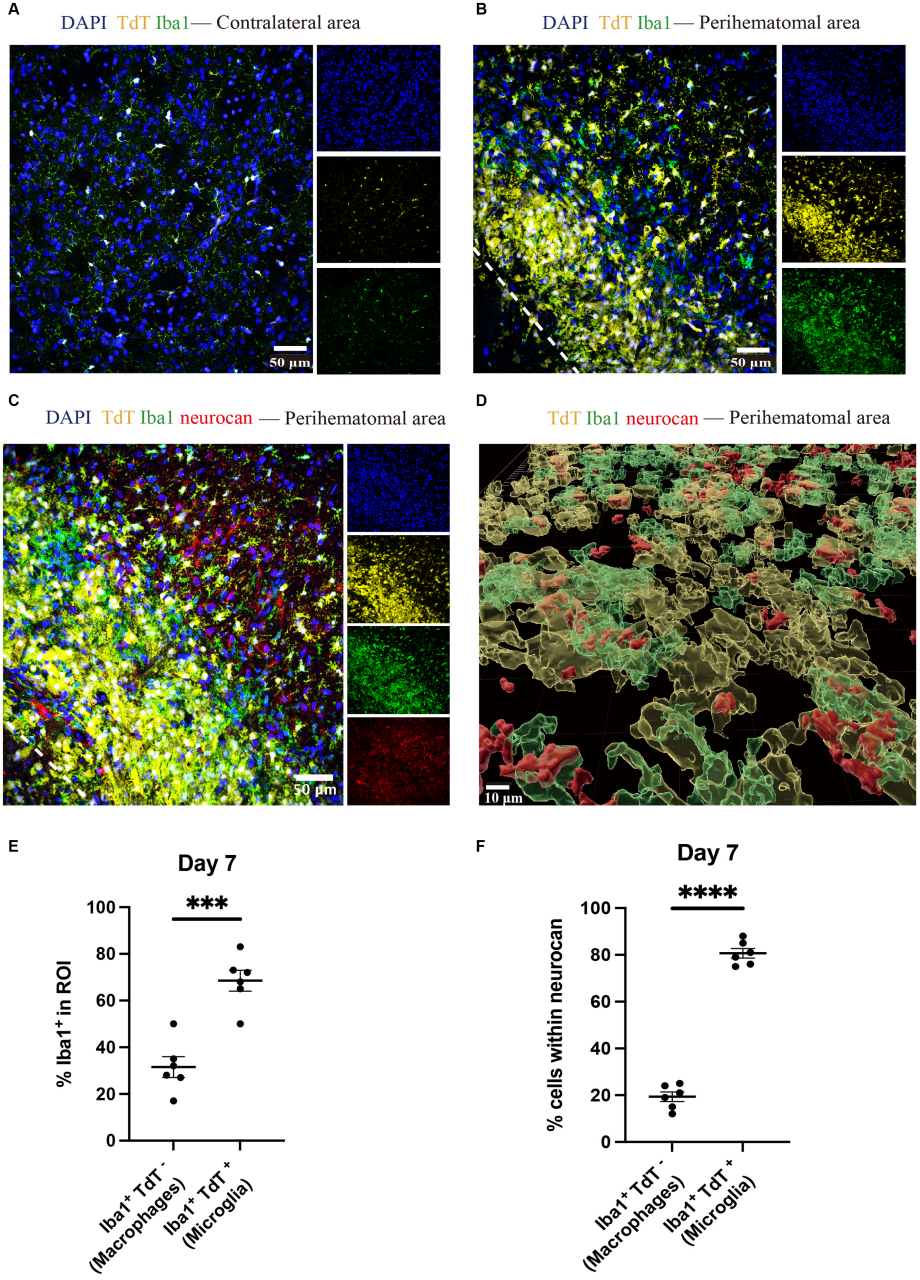
Microglia outnumber monocyte-derived macrophages in the perihematomal area in ICH mouse model Representative confocal images of brain sections from CX3CR1^CreER^:Rosa26^TdT^ ICH mice in contralateral area **(A)** and perihematomal **(B)** at day 7 stained for DAPI for cell nuclei (blue), Iba1 (green), TdT (yellow) and for neurocan (red) **(C)**. The lower left corner inside the dotted lines is the lesion core of ICH. Scale bar = 50 μm. **(D)** Imaris rendition of cells in the perihematomal area. Scale bar = 10 μm. **(E)** Quantification showing the percentage of Iba1^+^ in region of interest (ROI) that are TdT^−^ (i.e., monocyte-derived macrophages) or TdT^+^ (microglia). **(F)** More microglia than macrophages express neurocan in lesions. Data are presented as the mean ± SEM of 6 mice. Each dot represents mean of 4 locations of each area analyzed per mouse. Unpaired two-tailed Student’s *t*-test. Significance indicated as ***p < 0.001, ****p < 0.0001.

Brain tissues from tamoxifen-pretreated CX3CR1^CreER^:Rosa26^TdT^ mice with ICH were used for staining to distinguish microglia (Iba1 + TdT+) and monocyte-derived macrophages (Iba1 + TdT−) (Michaels et al., 2020; Plemel et al., 2020) in the perihematomal aera. Higher amounts of microglia were found in the perihematomal area at 7 days post-ICH than in the contralateral area (Figures 6A,B). Furthermore, the level of microglia was significantly higher than that of macrophages (Figure 6).

The ability to differentiate microglia from macrophages enabled us to address which of these myeloid populations preferentially were associated with intracellular neurocan. By enumerating Iba1+ TdT+ or TdT− cells, our data show that 80% of microglia were associated with neurocan while this was 20% for macrophages (Figures 6C–F).

### 3.5. OPCs are found in the perihematomal area of murine ICH

**Figure 7:**
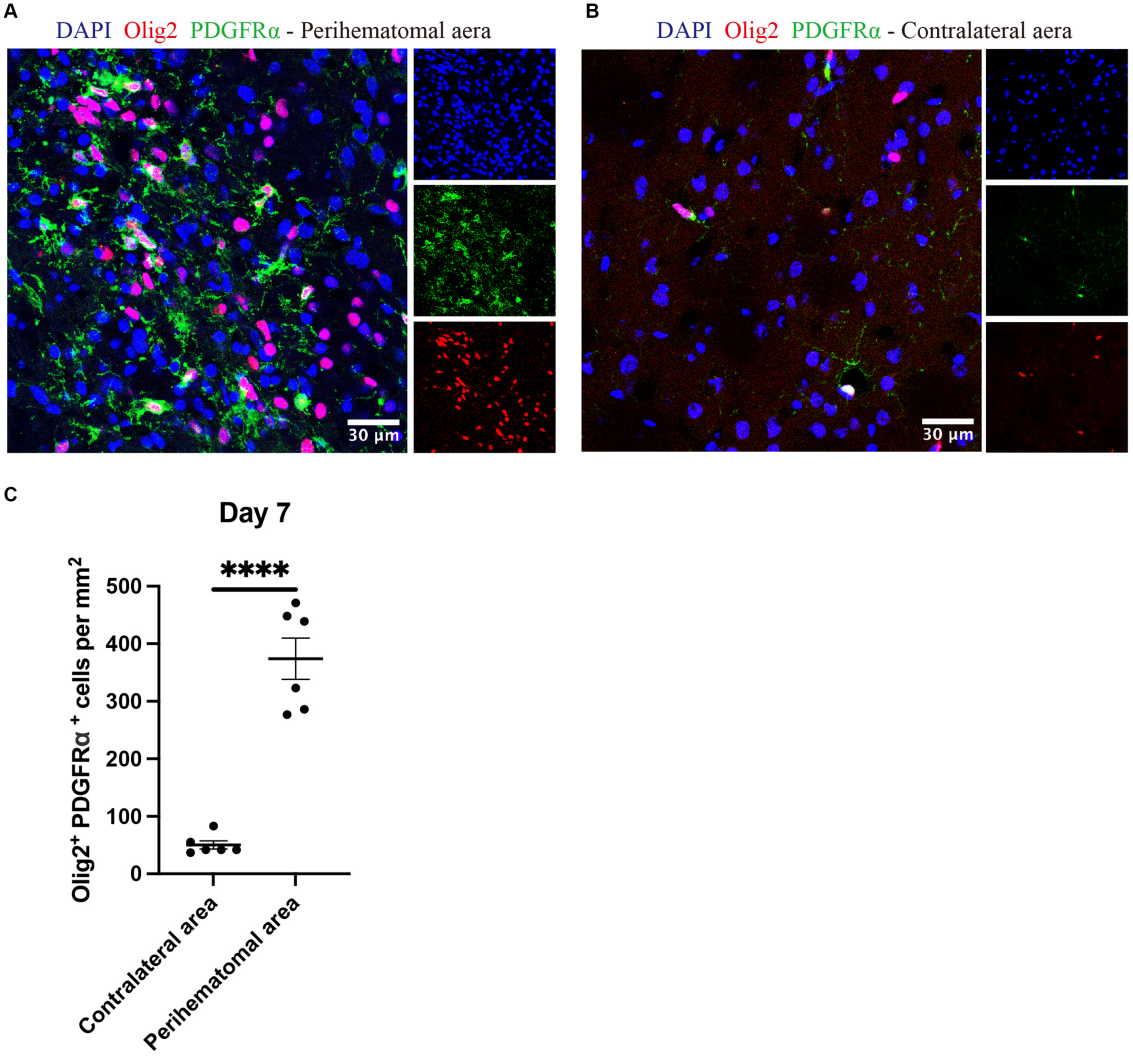
OPCs are elevated in perihematomal area of ICH lesions Representative confocal images of perihematomal area **(A)** and contralateral hemisphere **(B)** at day 7 in ICH stained for DAPI for cell nuclei (blue), Olig2 (red) and PDGFRα (green). Scale bar = 30 μm. **(C)** Bar graphs comparing the number of OPCs (olig2^+^ PDGFRα^+^); *N* = 6 replicates for each group, where each dot represents mean of 4 locations of each area (containing white and gray matter) analyzed per mouse. Data are presented as the mean ± SEM. Unpaired two-tailed Student’s *t*-test. Significance indicated as ****p < 0.0001.

OPCs identified by olig2^+^PDGFRα^+^ (Keough et al., 2016; Plemel et al., 2020) were noted in the perihematomal area containing both white and gray matter in expanded numbers than cell counts in the uninjured contralateral hemisphere (Figure 7). The co-presence of OPCs and neurocan in the perihematomal area suggest their potential for interactions which we next assessed in tissue culture.

### 3.6. Neurocan inhibits OPCs in culture

**Figure 8:**
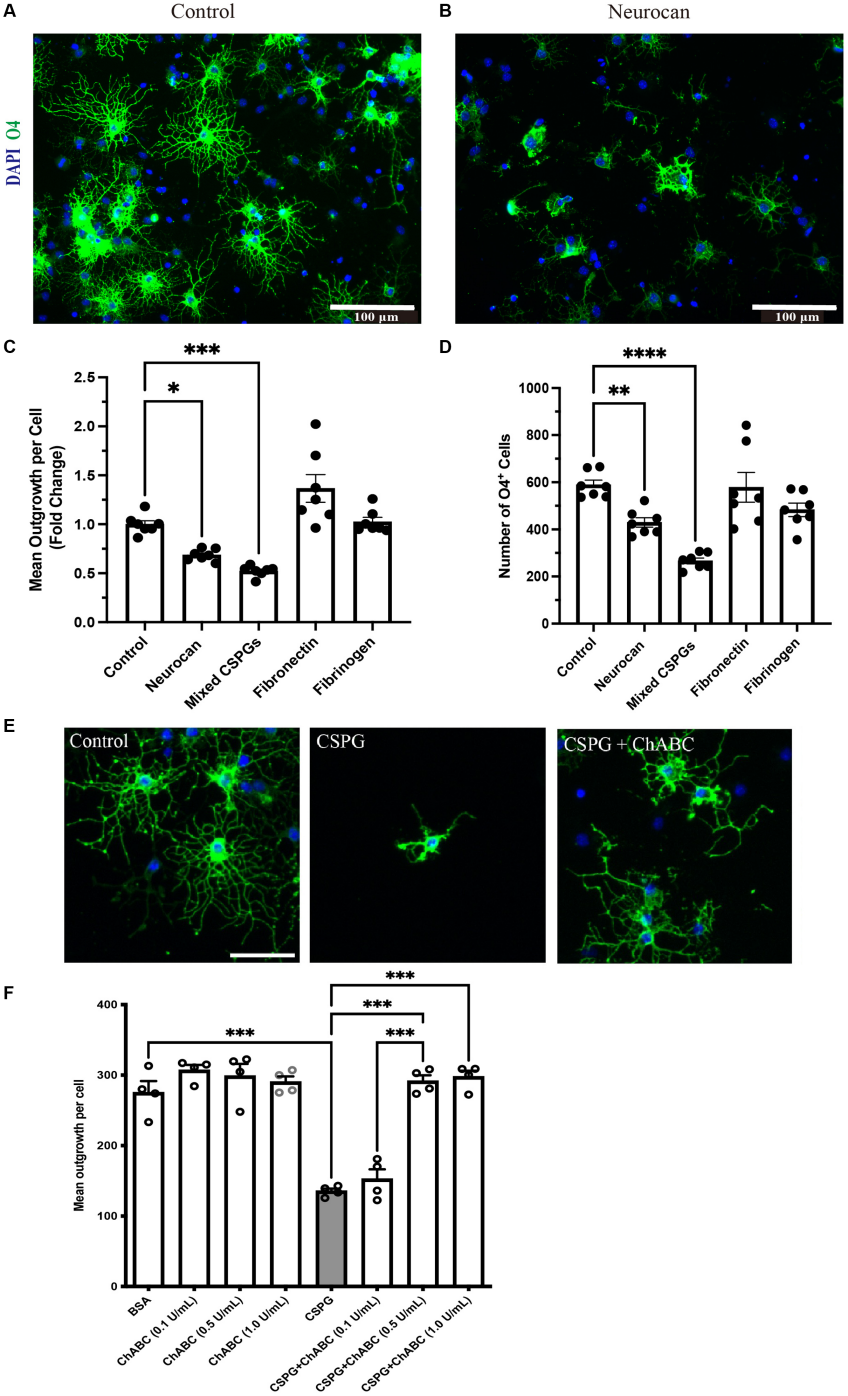
OPCs are inhibited by CSPGs and neurocan in culture Representative images of mouse OPCs stained for the sulfatide O4 24 h after plating onto PBS (control) **(A)** or neurocan **(B)** coated wells. Scale bar = 100 μm. Bar graphs comparing the fold change of mean process outgrowth of mouse OPCs **(C)**, or number of O4^+^ cells **(D)** cultured on control, neurocan, mixed CSPGs, fibronectin and fibrinogen after 24 h (*n* = 7 replicates). Data are presented as mean ± SEM. This was duplicated in two separate experiments. **(E,F)** The poor process outgrowth of OPCs when plated on CSPGs is overcome by ChABC at 0.5 and 1 U/mL. Each single data point represents average of 12 images (field of views, FOVs) of each well of cells. One-way ANOVA with Dunnett *post hoc*. *p < 0.05, **p < 0.01, ***p < 0.001, ****p < 0.0001.

Although CSPG molecules such as versicans and aggrecan can inhibit OPCs (Pendleton et al., 2013; Keough et al., 2016; Ghorbani et al., 2022), this is not known for neurocan. Given the prominence of neurocan expression in murine ICH, we evaluated its activity on OPCs. Figure 8 shows that when OPCs were seeded onto a substrate coated with neurocan, there were fewer O4+ OPCs that attached. Of the attached cells, these had fewer processes which, *in vivo*, are the precursors of myelin sheaths. Similarly, a mixed CSPG preparation used as a positive control also inhibited OPC adhesion and process outgrowth. Fibronectin and fibrinogen were tested but these did not affect OPCs (Figure 8). These results highlight that the neurocan elevation in murine could be a substantial impediment to OPCs that may attempt remyelination following ICH.

To address whether the GAGs or core protein of neurocan are responsible for inhibiting OPCs, we sought to incubate neurocan with ChABC which removes GAGs. However, the commercial vendor has since placed neurocan on indefinite backorder, so we used the mixed CSPG preparation which contains neurocan instead. Figures 8E,F show that ChABC overcomes the CSPG inhibition of OPC process outgrowth; thus, the GAGs of CSPGs and neurocan are the inhibitory moieties for the morphological differentiation of OPCs.

### 3.7. High levels of neurocan in human ICH

**Figure 9:**
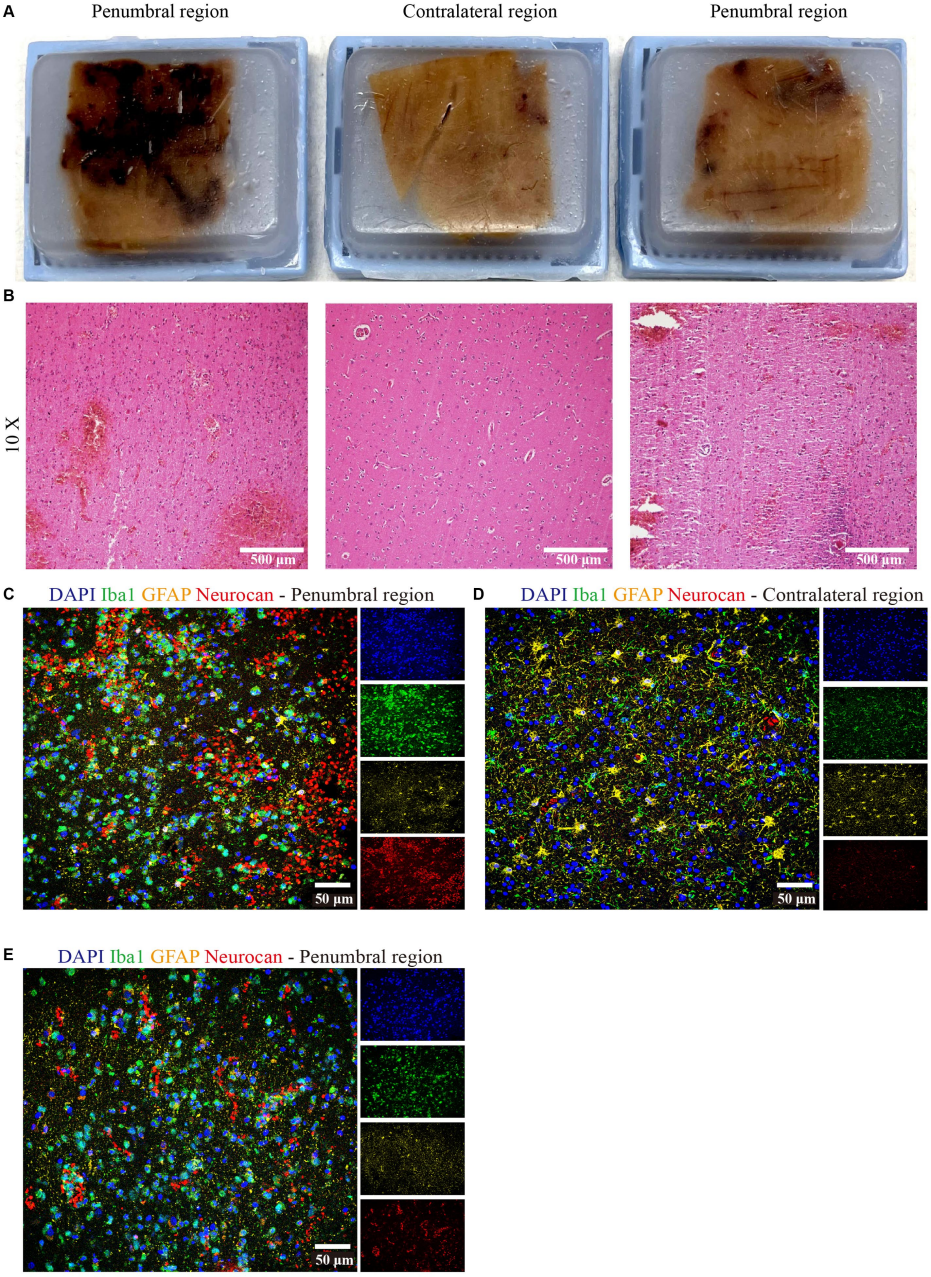
Neurocan is prominently increased in human ICH. **(A)** Human paraffin brain samples encompassing both hemorrhagic edge and perihematomal parenchyma regions, or the contralateral unaffected parenchyma, from a MCA infarct with hemorrhagic transformation. **(B)** Perihematomal and contralateral regions were stained with H&E to assess hemorrhage lesions. Scale bar = 500 μm. Representative immunofluorescent images of human brain tissue at different perihematomal **(C,E)** and contralateral **(D)** regions labeled with DAPI for cell nuclei (blue), Iba1 for microglia/macrophages (green), GFAP for astrocytes (yellow) and neurocan (red). Scale bar = 50 μm.

We had the opportunity to corroborate the mouse data using a case of human ICH. The central hemorrhage core consisted purely of necrotic tissue and coagulated hemorrhage and could not be analyzed. Two distinct penumbral and contralateral regions were analyzed. Tissue sections were taken from hemorrhage periphery and perihematomal parenchyma (Figure 9A), and the integrity of the tissue was informed by H&E staining (Figure 9B). The latter highlighted diffuse parenchymal injury and hemorrhage in the affected frontal white matter. Importantly, similar to murine data, neurocan was highly deposited in perihematomal regions, closely associated with Iba1^+^ microglia/macrophages (Figures 9C,E), as compared to contralateral region (Figure 9D).

## 4. Discussion

The poor prognosis of ICH is associated with mechanical disruption of brain by the enlarged hematoma and the progressive inflammatory responses (Wan et al., 2019; Bai et al., 2020; Xue and Yong, 2020). Hematoma size gradually decreased over time, with persistent activated microglia/macrophages and astrocytes however (Cao et al., 2016), especially in the perihematomal region (Supplementary Figures S1, S2). While these events are well recognized in ICH, the deposition of ECM molecules is a dearth of knowledge in stroke.

Deposited ECM components play a significant role in inflammatory outcomes following CNS injury (Stephenson et al., 2018). Findings from ischemic stroke studies have highlighted that specific ECM components including CSPGs, fibronectin, thrombospondin, tenascin-C and laminins are elevated and exacerbate injury by enhancing neuroinflammation in the perihematomal area (Jucker et al., 1993; Sakai et al., 2001; Li et al., 2012). Similarly, there is an upregulation of aggrecan, brevican, neurocan and versican in rabbit pups after intraventricular hemorrhage (Vinukonda et al., 2013). CSPGs are known drivers of neuroinflammation, as well as inhibitors of remyelination and axonal regeneration in the CNS (Lau et al., 2012; Keough et al., 2016). Tenascin-C is increased after subarachnoid hemorrhage and may cause cytotoxic and vasogenic edema (Midwood and Orend, 2009; Suzuki et al., 2020). Tenascin-C is found to be elevated after ICH in rats, and in the serum of patients with ICH (Wang et al., 2018; Ding et al., 2022). Overall, despite the pathologic significance of ECM, the expression and functions of ECM members in ICH have largely not been elucidated. Thus, we determined whether ECM molecules levels were altered in the collagenase-induced mouse model of ICH and in human ICH.

We found that neurocan was the predominant lectican CSPG in the perihematomal area and lesion core in ICH models, and expressed in perihematomal area of accumulated GFAP^+^ and Iba1^+^ cells (Figure 1), consistent with previous studies showing astrocytes and macrophages were the major producers of CSPGs following non-ICH injury in the CNS (Tang et al., 2003; Stephenson et al., 2018; Ghorbani et al., 2022). Neurocan was also highly accumulated in hemorrhagic and penumbral regions in human ICH (Figure 9). In contrast, the levels of aggrecan, versican-V1 and versican-V2 molecules were not upregulated in ICH (Figure 3). These results highlight the prominence of neurocan inside and around the hematoma in ICH. Strikingly, these results differ from those observed in EAE, with prominent versican-V1 but not neurocan elevation. Furthermore, TSP and TNC were not elevated in ICH while being highly expressed in EAE (Figure 3). The possible reason for the difference between EAE and ICH may be related to the inflammatory milieu. ICH is an acute brain injury condition where neutrophil accumulation is observed within 2 days and is still apparent at 12 days in patients with ICH (Shtaya et al., 2019). However, T lymphocytes are found in low level in the perihematomal area (Wimmer et al., 2018). In contrast, in EAE, autoreactive T and B cells play a prominent role in the immune response.

We also found fibrinogen, fibronectin and HSPG to be upregulated in the perihematomal area and lesion core, and their expression in the perihematomal area are within GFAP^+^ and Iba1^+^ cells (Figure 5). These findings are in accordance with other models in which fibronectin and HSPG molecules are expressed in CD45^+^ leukocytes, activated macrophages and reactive astrocytes following CNS injury (Milner et al., 2007; Ghorbani et al., 2022). Transforming growth factor beta and epidermal growth factor signaling pathways contribute to the upregulation of ECM molecules in astrocytes (Jahan and Hannila, 2015). The damage to the blood–brain barrier and deposition of plasma-derived ECM such as fibrinogen can also lead to their increase within the CNS parenchyma. These proteins have been shown to promote TGF-beta signaling in astrocytes, which can further stimulate the local production of ECM proteins (Schachtrup et al., 2010). We did not find tenascin-C to be increased in our ICH lesions at day 7 while staining was observed in the EAE lesion control. Our results are thus discrepant with those of others where tenascin-C was found to be elevated after ICH in rats, and in the serum of patients with ICH (Wang et al., 2018; Ding et al., 2022). Further work would need to be conducted to resolve this discrepancy.

A limitation of our study is that the association of staining of molecules within cells does not mean that the cell is producing the ECM of interest, as the cells may phagocytose deposited ECM and thus display intracellular immunoreactivity. Thus, we do not know if neurocan is synthesized and released by microglia and astrocytes. A future endeavor would be to conduct *in situ*-hybridization studies to ascertain the sources of neurocan mRNA with specific cell types after ICH injury.

We evaluated the levels of OPCs in the perihematomal area after ICH and noted that the density of OPCs significantly increased compared to the contralateral aera (Figure 7). This is consistent with a previous study showing that OPCs are upregulated in the perihematomal aera (Joseph et al., 2016). However, if neurocan is an inhibitor of OPC activity as our tissue culture results indicate (Figure 8), then it is possible that the injury-induced elevation of OPCs in the perihematomal area would be further enhanced if inhibitors to CSPG synthesis could be introduced as therapeutics. This is a direction for future studies.

We observed that mixed CSPGs exert a more potent inhibitory effect on process outgrowth and differentiation of OPCs than neurocan (Figure 8). One possible reason is that the CSPG mixture contains other lectican CSPG members including aggrecan, brevican and versican, which may bind to more receptors and act in synergy to inhibit OPCs (Matsuyama et al., 2022).

In summary, ICH is accompanied by elevation of several ECM molecules. One of these, neurocan, is a potent inhibitor of properties of OPCs *in vitro* and may hinder attempts at remyelination *in vivo*. The diversity of expressed ECM components in ICH suggests a multitude of functions in ICH that deserves future studies to define their distinct or collective roles in ICH. This study introduces the potential of targeting ECM as an untapped approach to improve the prognosis of ICH.

## Data availability statement

The datasets presented in this study can be found in online repositories. The names of the repository/repositories and accession number(s) can be found in the article/Supplementary material.

## Ethics statement

The studies involving humans were approved by the Conjoint Health Research Ethics Board at the University of Calgary (Ethics ID REB15-0444). The studies were conducted in accordance with the local legislation and institutional requirements. The human samples used in this study were acquired from Foothills Medical Center, University of Calgary. Written informed consent for participation was not required from the participants or the participants’ legal guardians/next of kin in accordance with the national legislation and institutional requirements. The animal study was approved by the Animal Care Committee at the University of Calgary. The study was conducted in accordance with the local legislation and institutional requirements.

## Author contributions

HL conceived the project, designed, performed and analyzed the experiments, wrote the first draft of the manuscript. SG and MK provided the cell culture data. RZ provided initial training for surgery and helped with image analysis and quantitation. VE helped with histology and confocal imaging. ES performed the human autopsy, characterized the lesional areas by histology, and provided human neuropathology oversight. VY and MX co-conceived the project, provided support, supervised the overall study, critically edited the manuscript, and were guarantors of the results. All authors reviewed and edited the manuscript.

## Abbreviations

CNS: central nervous system
CSPGs: chondroitin sulfate proteoglycans
EAE: experimental autoimmune encephalomyelitis
ECM: extracellular matrix; GAG: glycosaminoglycan
GFAP: glial fibrillary acidic protein
HSPGs: heparan sulfate proteoglycans
Iba1: Ionized calcium-binding adapter molecule 1
ICH: intracerebral hemorrhage
MCA: middle cerebral artery
MMPs: matrix metalloproteinases
OPC: oligodendrocyte precursor cells
PBS: Phosphate-buffered saline
PFA: Paraformaldehyde
TNC: tenascin-C
TSP: thrombospondin
