# Supplementary material for: Prominent elevation of extracellular matrix molecules in intracerebral hemorrhage
Source: Front Mol Neurosci. 2023 Nov 6;16:1251432. doi: 10.3389/fnmol.2023.1251432 (PMC10658787; doi:10.3389/fnmol.2023.1251432)
Supplement: Supplementary file 1 [file Presentation_1.pdf]

Supplementary Figure S1. ICH injury in mice induced by collagenase.

(A) Frozen brain sections from ICH mice at different time points were stained with H&E to identify lesions. Black rings indicate the hematoma sites. Scale bar = 1mm. **(B)** Representative images of coronal brain sections from ICH mice labeled with DAPI for cell nuclei (blue), Iba1 (yellow), and CD45 (green), with Imaris rendering shown in **C**. (**D**, **E**) Representative images of coronal brain sections from ICH mice labeled for GFAP (grey) and NeuN (red) showing the site of the perihematomal area (**E**). The lower left corner within the dotted lines is the lesion core.

Supplementary Figure S2. Isotype control and secondary antibody control of neurocan staining.

(A) Representative confocal images of brain sections from ICH mice at perihematomal area, lesion core and contralateral area at day 7 stained with DAPI for cell nuclei (blue), Iba1 (green) and neurocan (red). **(B)** Representative confocal images of brain sections from ICH mice at perihematomal area, lesion core and contralateral area at day 7 stained with DAPI for cell nuclei (blue), Iba1 (green) and isotype antibody (red). **(C)** Representative confocal images of brain sections from ICH mice at perihematomal area, lesion core and contralateral area at day 7 stained with only secondary antibodies and DAPI (blue). The lower left corner within the dotted lines is the lesion core. Scale bar = 50 μm.
