# Supplementary figures and images for: Prominent elevation of extracellular matrix molecules in intracerebral hemorrhage
Source: Front Mol Neurosci. 2023 Nov 6;16:1251432. doi: 10.3389/fnmol.2023.1251432 (PMC10658787; doi:10.3389/fnmol.2023.1251432)

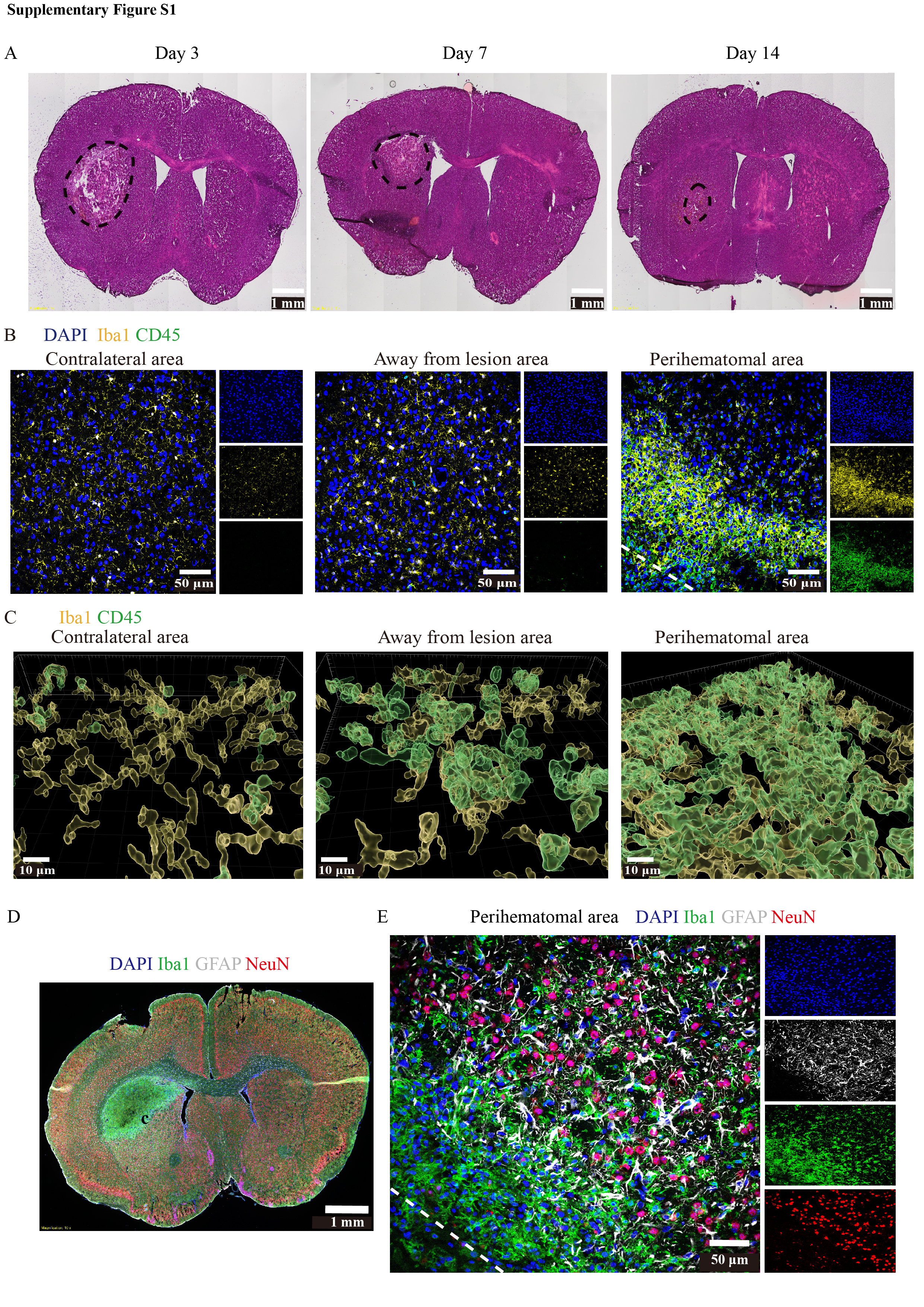

Supplement: Supplementary file 2 [file Image_1.JPEG]

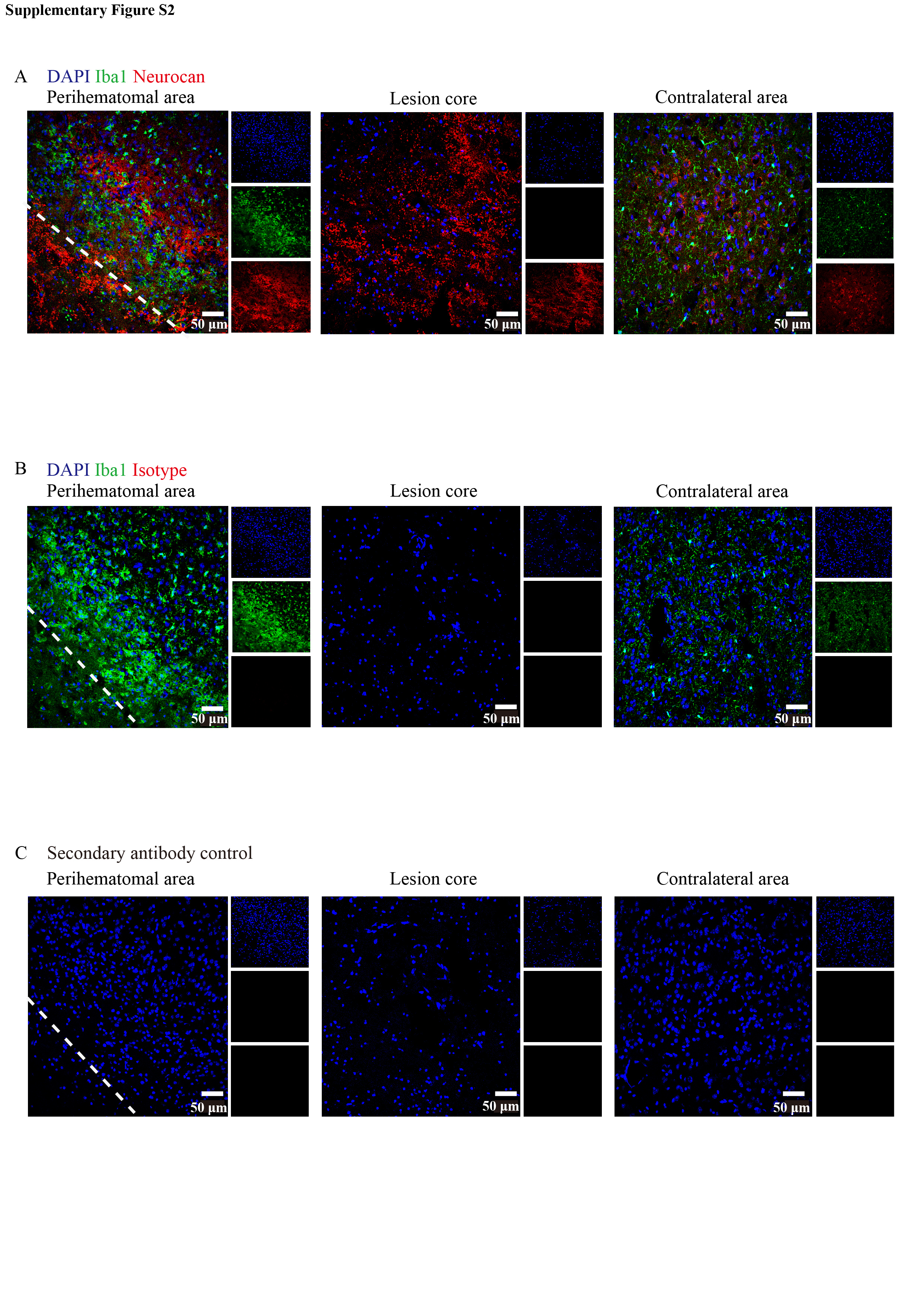

Supplement: Supplementary file 3 [file Image_2.JPEG]
